# Structural Basis for the Shared Neutralization Mechanism of Three Classes of Human Papillomavirus Type 58 Antibodies with Disparate Modes of Binding

**DOI:** 10.1128/JVI.01587-20

**Published:** 2021-03-10

**Authors:** Maozhou He, Xin Chi, Zhenghui Zha, Yunbing Li, Jie Chen, Yang Huang, Shiwen Huang, Miao Yu, Zhiping Wang, Shuo Song, Xinlin Liu, Shuangping Wei, Zekai Li, Tingting Li, Yingbin Wang, Hai Yu, Qinjian Zhao, Jun Zhang, Qingbing Zheng, Ying Gu, Shaowei Li, Ningshao Xia

**Affiliations:** a State Key Laboratory of Molecular Vaccinology and Molecular Diagnostics, School of Public Health, School of Life Sciences, Xiamen University, Xiamen, China; b National Institute of Diagnostics and Vaccine Development in Infectious Disease, Xiamen University, Xiamen, China; c Research Unit of Frontier Technology of Structural Vaccinology, Chinese Academy of Medical Sciences, Xiamen, China; International Centre for Genetic Engineering and Biotechnology

**Keywords:** human papillomavirus type 58, cryo-EM structure, neutralization mechanism, neutralizing antibody

## Abstract

Human papillomavirus type 58 (HPV58) is associated with cervical cancer and poses a significant health burden worldwide. Although the commercial 9-valent HPV vaccine covers HPV58, the structural and molecular-level neutralization sites of the HPV58 complete virion are not fully understood. Here, we report the high-resolution (∼3.5-Å) structure of the complete HPV58 pseudovirus (PsV58), determined by using cryo-electron microscopy (cryo-EM). Three representative neutralizing monoclonal antibodies (nAbs 5G9, 2H3, and A4B4) were selected through clustering from a panel of nAbs against HPV58. Bypassing the steric hindrance and symmetry mismatch in the HPV Fab-capsid immune complex, we present three different neutralizing epitopes in the PsV58 and show that, despite differences in binding, these nAbs share a neutralization mechanism. These results offer insight into HPV58 genotype specificity and broaden our understanding of HPV58 neutralization sites for antiviral research.

**IMPORTANCE** Cervical cancer primarily results from persistent infection with high-risk types of human papillomavirus (HPV). HPV type 58 (HPV58) is an important causative agent, especially in Asia. Despite this, we still have limited data pertaining to the structural and neutralizing epitopes of HPV58, and this encumbers our in-depth understanding of the virus’ mode of infection. Here, we show that representative nAbs (5G9, 10B11, 2H3, 5H2, and A4B4) from three different groups share a neutralization mechanism that appears to prohibit the virus from associating with the extracellular matrix and cell surface. Furthermore, we determined that the nAbs engage via three different binding patterns: top-center binding (5G9 and 10B11), top-fringe binding (2H3 and 5H2), and fringe binding (A4B4). Our work shows that, despite differences in the pattern in binding, nAbs against HPV58 share a neutralization mechanism. These results provide new insight into HPV58 infection.

## Abstract (precis)

Cervical cancer primarily results from persistent infection with high-risk types of human papillomavirus (HPV). HPV type 58 (HPV58) is an important causative agent, especially in Asia.

## INTRODUCTION

High-risk human papillomavirus (HPV) infection causes several types of epithelial tumors, particularly cervical cancer, the leading cause of death among women across the globe (1, 2). More than 18 types of genital HPV (including HPV16 and HPV58) are classified as high risk because of their epidemiological link with cervical cancer (3). Remarkably, in Asia and some American countries, HPV58 ranks third among the cause of cervical cancer cases, although globally, it contributes to only about 3.3% of cases (4). In particular, HPV58 is frequently detected among Korean women with abnormal cytology (5) and is associated with all grades of lesions among women in Mexico, sometimes at levels higher than that seen for HPV16 (4).

As has been widely reported, HPV virions are composed of the major (L1) and minor (L2) capsid proteins, which surround the 8-kb closed, circular DNA genome to form a T=7 icosahedral capsid ∼60 nm in diameter (6). Five copies of the L1 protein are intertwined to form a capsomer (or a pentamer), with 72 capsomers undergoing self-assembly to form one capsid, also termed a virus-like particle (VLP), that is composed of L1 only. The L2 protein is not required for the formation of an intact capsid shell (7). Among 72 capsomers, 12 capsomers along the icosahedral 5-fold vertex are referred to as pentavalent capsomers, whereas the others are described as hexavalent capsomers. Each L1 structure comprises eight antiparallel beta-strands (BIDG and CHEF) in the form of a jelly roll structural motif which is maintained by connections among the BC, DE, EF, FG, and HI surface loops (8). The C terminus of each HPV L1 capsid protein extends along the capsid canyon to link with the adjacent capsomer and back to the original donor capsomer; this region, also referred to as the C-terminal arm, is consistent with the invading arm structure of the bovine papillomavirus (9–11). We previously reported the crystal structure of the HPV58 pentamer (12); however, there is still no high-resolution structure of the complete HPV58 virion. Such a structure would help to clarify intercapsomer interactions and elucidate differences between HPV58 and other common subtypes, such as HPV16.

Neutralizing antibodies (nAbs) are common tools for analyzing virus epitopes and infection mechanisms. To date, several representative nAbs (e.g., HPV16.V5 and HPV16.U4) have been generated and are extensively used in HPV vaccine research and related serological assays (13, 14). Biochemical and structural analyses have mapped the neutralizing binding sites for HPV6, -11, -16, -31, -33, -52, -58, and -59 to the hypervariable surface loops BC, DE, EF, FG, and HI of the L1 major capsid protein, as well as the C-terminal arm (8, 11, 12, 15–17). For HPV58, although several antibody panels have been developed (18), detailed information on the neutralization sites remain unknown. nAbs tend to neutralize via one of two classical neutralizing mechanisms: the first, as represented by HPV16.U4, blocks the cell surface association but allows extracellular matrix (ECM) binding; the other, as exemplified by HPV16.V5 and HPV16.E70, permits the cell surface association but blocks ECM binding (19). In addition, cryo-electron microscopy (cryo-EM) techniques have long been a powerful tool for analyzing the pattern of nAb binding to virus (20–23). Interestingly, cryo-EM structures of these immune complexes show that these two types of nAbs engage via different binding patterns (13, 14). However, the binding patterns and neutralizing behaviors of nAbs for other types of HPV remain unknown.

Here, we generated a panel of nAbs against HPV58 and clustered the antibodies into three groups. Immunofluorescence assays revealed that the five representative nAbs selected from the panel (5G9, 10B11, 2H3, 5H2, and A4B4) share a neutralizing mechanism. Further, cryo-EM analysis showed that these five nAbs can be subclassified into three groups with three distinct Fab binding patterns against Psv58. Combining the data from the subparticle reconstruction with the three-dimensional (3D) classification, we acquired high-resolution structures of Psv58 and well-defined Fab features in the Fab-capsid interaction, which allowed us to elucidate detailed epitope information for the three binding patterns. Collectively, these findings shed light on the structural basis of HPV58 type specificity and help to unravel the molecular details of HPV neutralization.

## RESULTS

### Generation and characterization of a panel of anti-HPV58 nAbs.

Purified HPV58 virus-like particles (VLPs) were used for immunization and to generate a panel of 15 anti-HPV58 nAbs, according to traditional methods, as detailed in Materials and Methods (Table 1). The isotypes of the 15 nAbs are IgG1 (2H3, 4A2, 5H2, 9G4, and A1H6), IgG2a (5G9, A8B1, and A4B4), and IgG2b (1D4, 3E4, 10B11, 11C7, 13A4, A6E7, and A10B8). A4B4 recognizes a linear epitope of HPV58 by Western blotting, whereas the other 14 nAbs recognize conformational epitopes. The activities of binding of the nAbs to HPV58 VLPs were then tested by indirect enzyme-linked immunosorbent assay (ELISA). The half-maximal effective concentrations (EC_50_s) for all 15 nAbs ranged from 42.47 ng/ml to 126.10 ng/ml. nAb 13A4 had the lowest EC_50_ (42.47 ng/ml), indicative of the highest binding activity with HPV58 VLPs. Through a neutralization assay using 2-fold dilution and a starting concentration of 1 μg/ml, we showed that all nAbs could neutralize PsV58 and prevent it from infecting cells, with neutralization titers ranging from 12,800 to 409,600. To evaluate the immunodominance of the epitopes recognized by these nAbs, a rabbit antiserum-based competitive ELISA was performed. We found that nAb 2H3 had the highest inhibition rate (87%) in its recognition of an immunodominant epitope.

**TABLE 1:** Characteristics of a panel of anti-HPV58 monoclonal antibodies

nAb	Isotype	Epitope type^*a*^	Binding affinity EC_50_ (ng/ml)^*b*^	Neutralization titer^*c*^	% epitope immunodominance (inhibition rate)^*d*^
1D4	IgG2b	C	80.66	204,800.0	51
2H3	IgG1	C	96.44	51,200.0	87
3E4	IgG2b	C	126.10	146,286.0	46
4A2	IgG1	C	96.57	29,767.4	36
5H2	IgG1	C	47.35	29,425.3	58
5G9	IgG2a	C	48.93	409,600.0	59
9G4	IgG1	C	42.68	12,800.0	60
10B11	IgG2b	C	58.54	102,400.0	42
11C7	IgG2b	C	63.54	102,400.0	59
13A4	IgG2b	C	42.47	12,800.0	53
A1H6	IgG1	C	45.54	25,600.0	61
A6E7	IgG2b	C	80.28	51,200.0	7
A8B1	IgG2a	C	116.70	25,600.0	31
A10B8	IgG2b	C	70.30	102,400.0	21
A4B4	IgG2a	L	108.50	12,800.0	71

a Epitope types were determined via Western blotting, C, conformational; L, linear.

b EC_50_s were derived from indirect binding ELISA data.

c Neutralization titers were determined by pseudovirus-based neutralization assay, where the concentration of nAb in the first well was 1 μg/ml, with subsequent 2-fold dilution.

d Inhibition rates of nAb against rabbit antiserum were calculated via inhibition ELISA.

**FIG 1:**
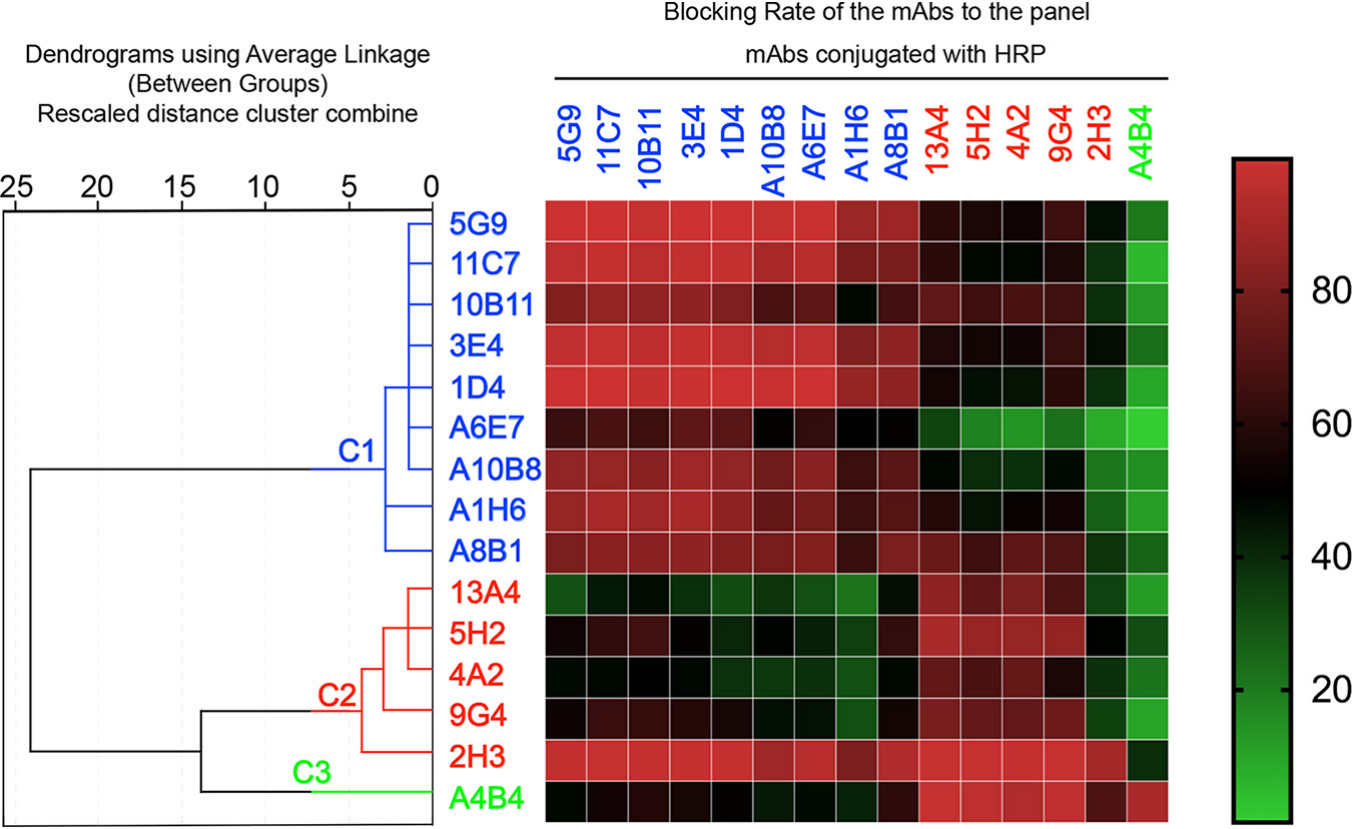
Clustering analysis of 15 nAbs directed against HPV58 VLPs based on competitive ELISA (cELISA). The 15 nAbs were clustered into 3 groups (C1 [blue], C2 [red], and C3 [green]) (left) using SPSS based on their cELISA profiles (right). The heat map of the cELISA data is depicted according to the inhibition rate (percent). Parameters are colored continuously from green to red (0, green; 50, black; 100, red).

To comprehensively understand the spatial configuration of the epitopes recognized by these nAbs, we performed a pairwise competitive ELISA (cELISA) with all 15 nAbs against HPV58 VLPs using conventional methods (24, 25). A heat map was generated based on the 15-by-15 cELISA data for SPSS clustering analysis (Fig. 1). Data were organized in an accurate and straightforward manner to classify nAb epitopes, and these results are depicted in the dendrogram plot (Fig. 1). Combining distance values with the heat map, we classified nAbs with distance values of <5 in the same group. Consequently, 15 nAbs were clustered into one of three groups, indicative of three relatively distinct epitopes: C1 (5G9, 11C7, 10B11, 3E4, 1D4, A6E7, A10B8, A1H6, and A8B1), C2 (13A4, 5H2, 4A2, 9G4, and 2H3), and C3 (A4B4). Notably, nAb 2H3, which recognizes an immunodominant epitope, showed the highest blocking rates among the nAbs, with average values of ∼60%. Only nAb A4B4 was classified in group C3, suggesting that it may recognize a unique epitope of HPV58.

### Potential neutralization mechanisms of the representative nAbs against HPV58.

**FIG 2:**
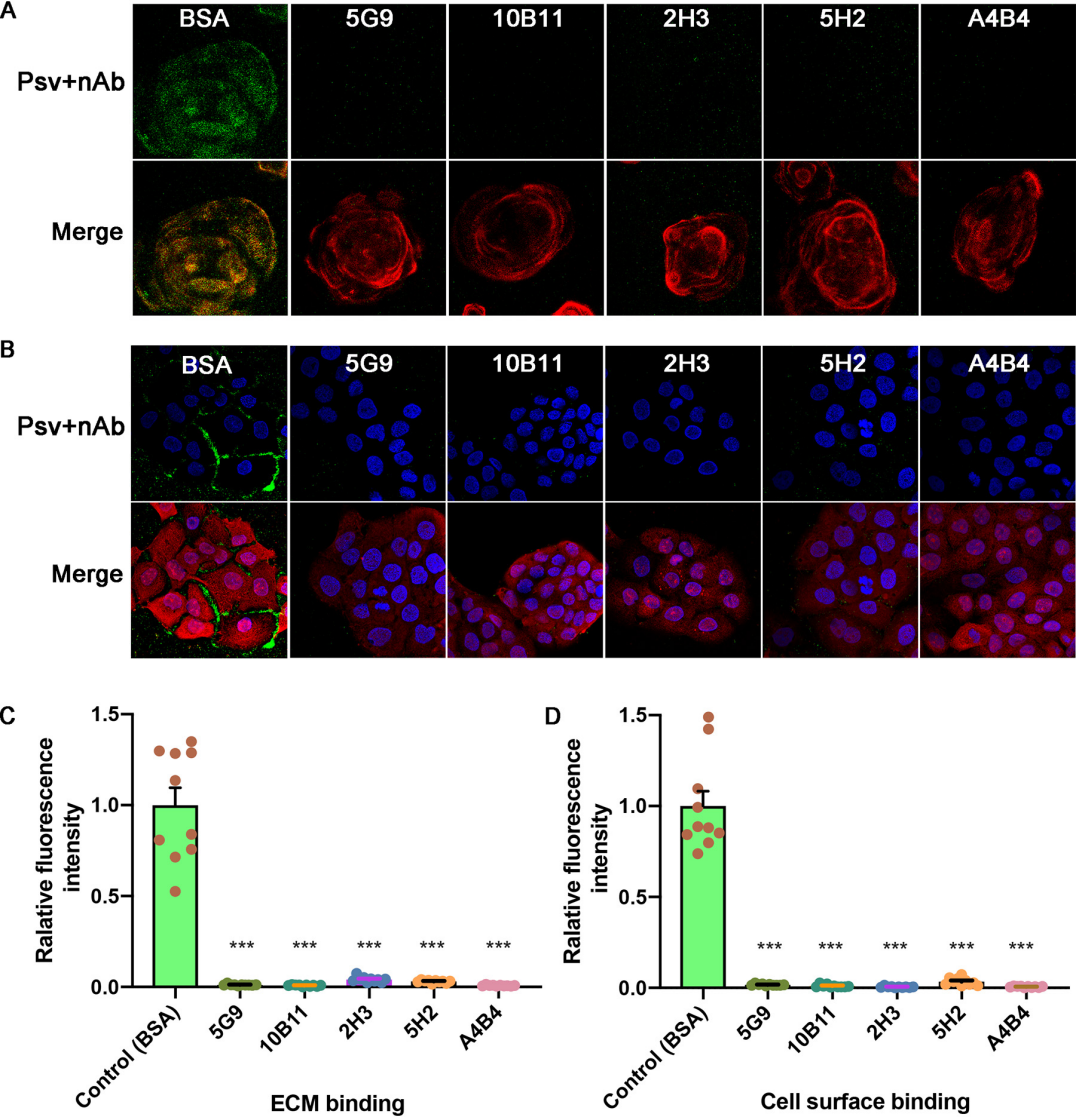
Attachment assay for HPV58 nAbs to the ECM and cell surface. Alexa Fluor 488-conjugated (green) PsV58 was preincubated with either BSA (control group) or 5G9, 10B11, 2H3, 5H2, and A4B4, as indicated, and then added to the ECM or the cell surface. Laminin-5 (red) stain was used for the ECM in an ECM binding assay (A), and phalloidin (red) was used for activity in the cell-surface binding assay (B). DAPI fluorescence (blue) identifies the cell nuclei (B). Green staining for PsV58 is shown in the upper panels, and the merged images are in the lower panels. (C) Intensities were calculated by randomly selecting 10 views using ImageJ, and the relative fluorescence in the ECM or cell surface infected with PsV58 is plotted as the mean and standard deviation (SD) against the BSA control group. Results were analyzed by unpaired Student's *t* test. ***, *P* ≤ 0.0001.

nAbs can function at different stages of the viral infection process, e.g., at attachment, during virus entry, or at various stages of intracellular activity (26). Previous observations show that HPV16 infection can be inhibited by binding of nAbs V5 and U4 to the extracellular matrix (ECM) or cell surface (19). Here, we performed an immunofluorescence assay to explore the neutralization mechanisms of five representative nAbs (5G9, 10B11, 2H3, 5H2, and A4B4) in HaCaT cells, selected from three different groups based on their binding and neutralizing efficacies. PsV binding to the ECM or cell surface was detected and quantified using fluorescence, with bovine serum albumin (BSA) used as a negative control. In the ECM attachment assay, there were high levels of green fluorescence in the BSA control group and almost no green fluorescence in the presence of any of the nAbs. This suggests that all 5 nAbs can block the binding of PsV58 to the ECM (Fig. 2A). The same blocking profile was noted for cell surface attachment (Fig. 2B). In 10 randomized microscopy inspection views, we calculated the mean fluorescence of PsV attachment and noted a significant difference between the control and nAb groups (*P* < 0.0001) (Fig. 2C). From these results, we assume that the representative nAbs from the three different groups share a neutralization mechanism against HPV58.

### Overall structure of the PsV58.

**FIG 3:**
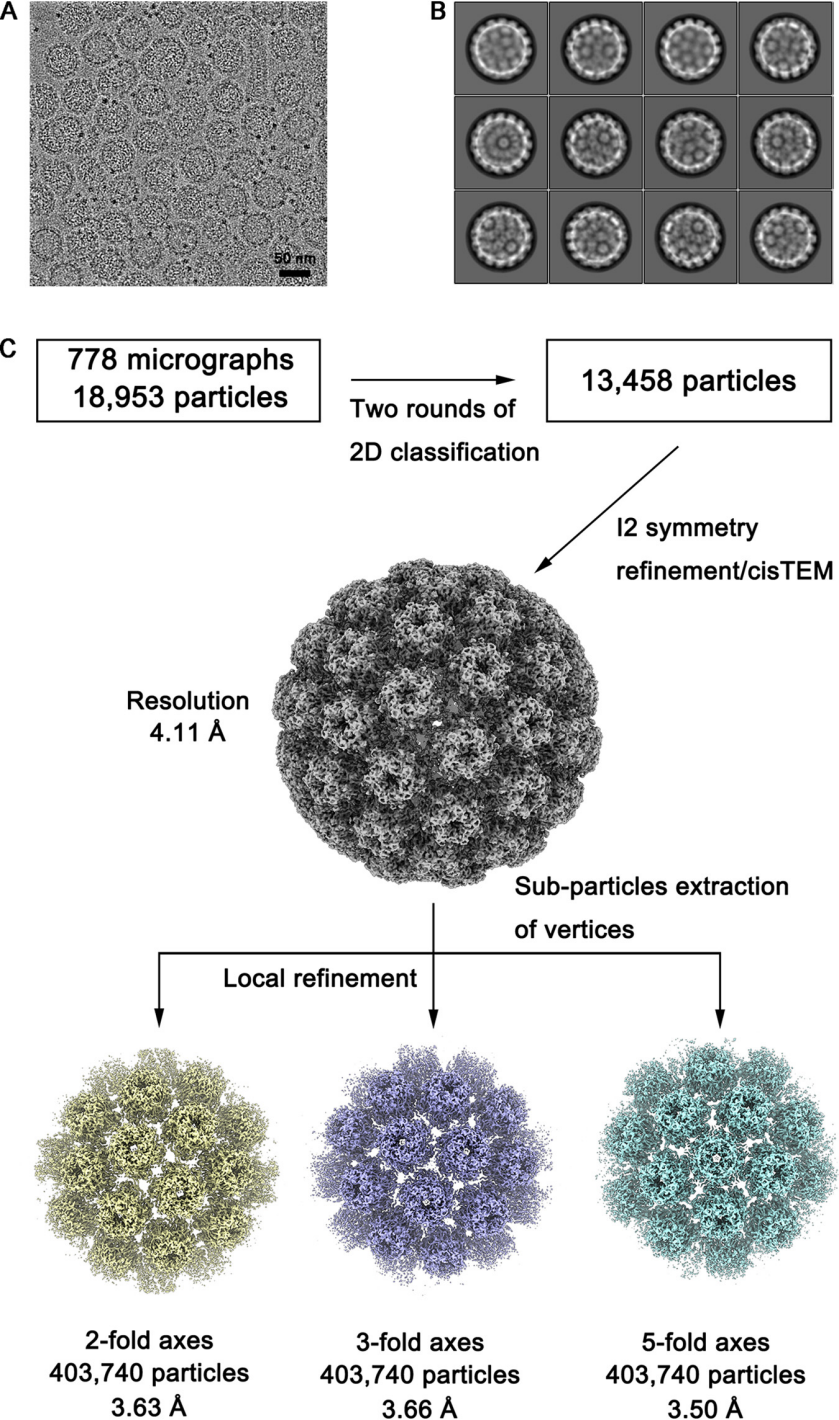
Cryo-EM reconstruction of the PsV58 capsid. (A) Representative cryo-EM micrograph of PsV58. Bar, 50 nm. (B) Representative 2D class averages of PsV58 particles as calculated with RELION. (C) Data processing schematic for icosahedral and subparticle reconstructions of the PsV58 capsid.

**FIG 4:**
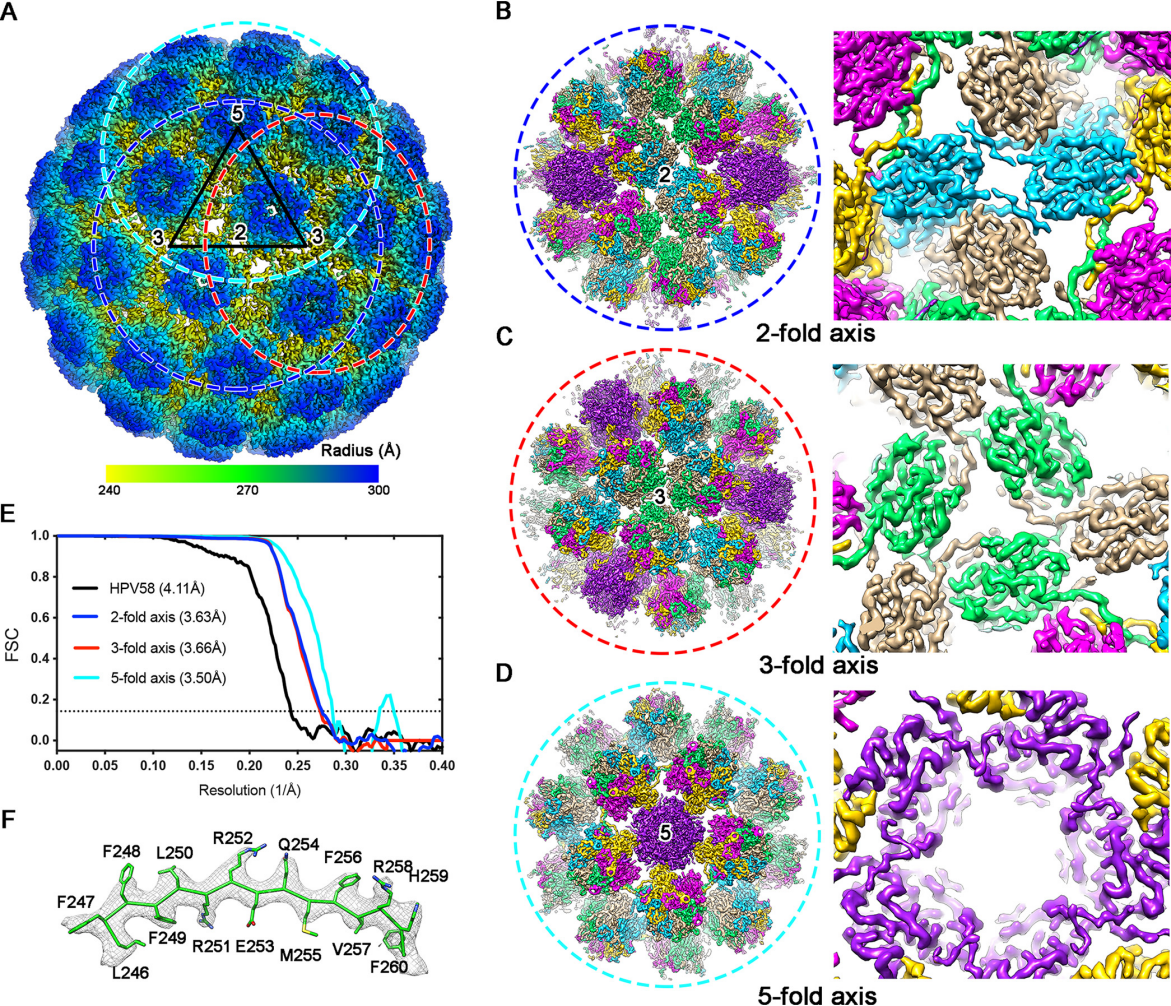
Structural analysis of the PsV58 capsid. (A) Isocontoured views (along the icosahedral 2-fold axis) of the cryo-EM map of PsV58 (radially colored from 240 Å to 300 Å) at a resolution of 4.11 Å. One icosahedral asymmetric unit is marked by a black triangle. The circles indicate the regions surrounding the 2-fold, 3-fold, and 5-fold axes (referred to as subparticles) and are colored blue, red, and cyan, respectively. The subparticles of the 2-fold (B), 3-fold (C), and 5-fold (D) vertexes were re-extracted and refined to improved resolutions of 3.63 Å, 3.66 Å, and 3.50 Å, respectively. Close-up views of these structures (from inside to outside) indicate the well-resolved side chain densities (B to D, right panels). The color scheme demonstrates the presence of six L1 monomers in one asymmetric unit. (E) FSC curves of the PsV58 capsid (black line) and subparticle reconstruction of its 2-fold axis (blue line), 3-fold axis (red line), and 2-fold axis (cyan line). (F) The atomic model of L1 monomer (residues L246 to F260, stick in green) is well fitted into the segmented density map (mesh in gray).

We next performed cryo-EM single-particle analysis to further explore the neutralizing epitopes targeted by the representative nAbs. Given the heterogeneity of HPV58 VLPs, which can drastically limit high-resolution structure determination, we opted to use PsV58 particles for further structural analysis. We purified a high concentration of PsV58 and vitrified the samples in holey carbon grids for cryo-EM data acquisition. The cryo-EM raw image showed slight heterogeneity of the PsV58 particles in terms of size (Fig. 3A). The 2D average results showed that most of the HPV58 sample had a diameter of ∼590 Å (Fig. 3B). Using a total of 13,458 selected particles, we reconstructed and determined the structure of the PsV58 at a resolution of 4.11 Å by the gold standard Fourier shell correlations (FSC = 0.143) with icosahedral symmetry using cisTEM software (Fig. 4A and E; Table 2) (27, 28). As expected, the HPV58 capsid comprises 72 capsomers arranged in a T=7d icosahedral lattice, with prominent “suspended bridges” between two neighboring capsomers. Although PsV58 was constituted with L1 and L2 by the expression of transfected plasmids carrying their genes, we did not observe any meaningful density that might be ascribed to L2, which is consistent with the cryo-EM structure reconstruction of the HPV16 quasivirus (QV) that is presumed to bear both L1 and L2 (EMD-6620) (29).

**TABLE 2:** Cryo-EM data collection, refinement and validation statistics

Characteristic	HPV58	HPV58-2fold	HPV58-3fold	HPV58-5fold	HPV58:5G9	HPV58:5G9-2fold	HPV58:10B11	HPV58:2H3	HPV58:2H3-2fold	HPV58:5H2	HPV58:A4B4	HPV58:A4B4-2fold
Data collection and processing												
Magnification	×93,000	×93,000	×93,000	×93,000	×93,000	×93,000	×78,000	×93,000	×93,000	×93,000	×93,000	×93,000
Voltage (kV)	300	300	300	300	300	300	300	300	300	300	300	300
Electron exposure (e^−^/Å^2^)	30	30	30	30	40	40	40	40	40	40	40	40
Defocus range (μm)	−1.7 to −4.4				−0.5 to −4.1		−0.9 to −4.5	−0.8 to −3.0		−1.1 to −5.0	−0.9 to −4.7	
Pixel size (Å)	1.128				1.121		1.327	1.121		1.121	1.121	
Symmetry imposed	I2	C2	C3	C5	I2	C1	I2	I2	C2	I2	I2	C2
Initial particle images (no.)	18,953				11,075		14,241	13,870		11,828	7,385	
Final particle images (no.)	13,458	403,740	269,160	161,496	5,276	14,464	1,599	8,710	104,213	8,166	7,181	94,862
Map resolution (Å)	4.11	3.63	3.66	3.50	4.67	6.41	6.43	4.08	3.64	4.33	4.23	4.19

Refinement												
Map-sharpening B factor (Å^2^)	−150	−90	−90	−90	−203	−150	−150	−175	−90	−179	−201	−90
Model composition												
Nonhydrogen atoms	22,115					21,764			21,792			21,853
Protein residues	2,787					2,761			2,762			2,769
RMS deviations												
Bond lengths (Å)	0.012					0.006			0.009			0.006
Bond angles (°)	1.410					1.294			1.291			1.239
Validation												
MolProbity score	2.01					2.07			1.88			1.83
Clashscore	6.55					11.72			6.18			6.60
Poor rotamers (%)	1.05					0.37			0.82			0.61
Ramachandran plot												
Favored (%)	86.27					92.06			90.21			92.60
Allowed (%)	13.51					7.72			9.61			7.30
Disallowed (%)	0.22					0.22			0.18			0.11

**FIG 5:**
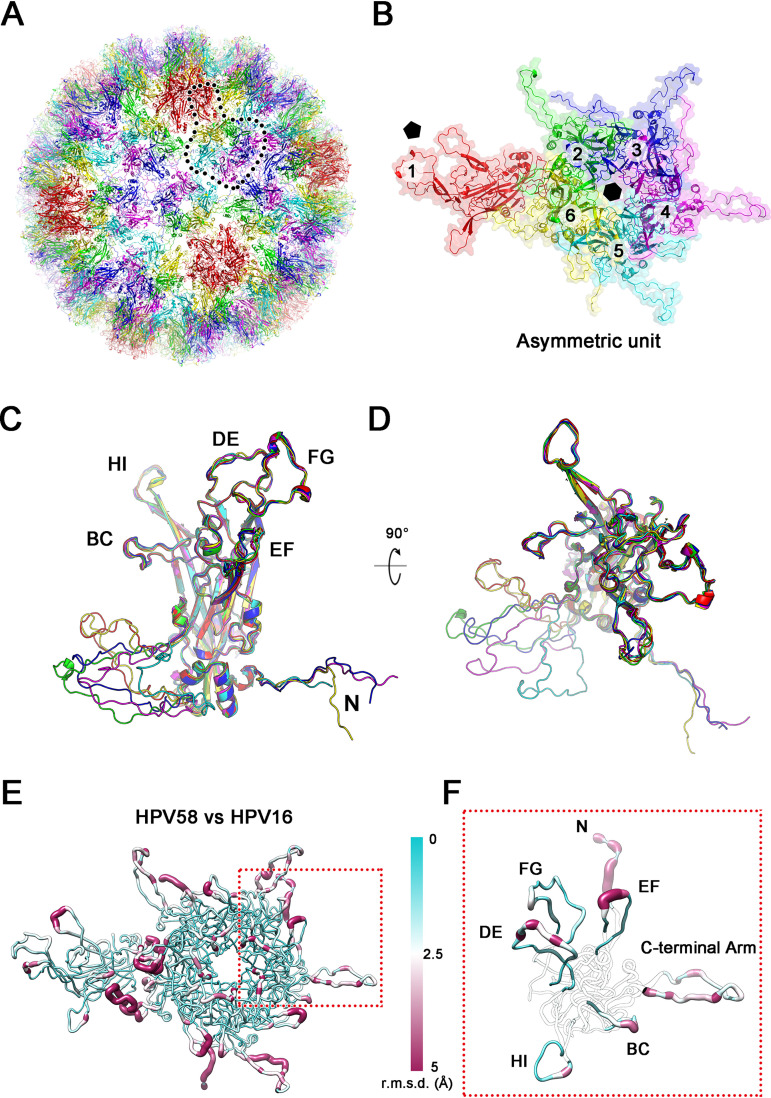
Atomic model of the PsV58 capsid. (A) Complete atomic model of HPV58 depicted as a ribbon diagram and viewed along the 2-fold axis. Color scheme of the six L1 monomers in the asymmetric unit: chain 1 (red), chain 2 (green), chain 3 (blue), chain 4 (magenta), chain 5 (cyan), and chain 6 (yellow). This color scheme is used throughout unless otherwise noted. (B) Ribbon diagram and surface presentations of the closeup view of an asymmetric unit. The black pentagon and hexagon mark the 5-coordinated and 6-coordinated pentamers of the capsid, respectively. (C and D) Superposition of the L1 monomers in one asymmetric unit of the PsV58 as viewed from the side (C) and the top (D). Differences between L1 models stem from the N and C termini. (E and F) The asymmetric unit of HPV58 is rendered according to the RMSD values, which range from 0 Å to 5 Å compared with HPV16. Line thickness represents RMSD variations in a worm diagram. (F) Close-up view of the structure in panel E shows RMSD variation in the regions of the surface loops (BC, DE, EF, FG, and HI loops) of chain 4. The other regions are transparent for ease of viewing.

The density map of the 4.11-Å-resolution structure is insufficient for modeling, particularly the flexible surface loops. Thus, to increase the resolution and overcome the issues with sample heterogeneity, we employed localized reconstruction to further refine the structure (30). Subparticles with a box size of ∼474 Å were separately extracted from the 2-fold, 3-fold, and 5-fold symmetric regions and were refined to 3.63 Å, 3.66 Å, and 3.50 Å, respectively (Fig. 4E and 3C; Table 2). Of note, this significant improvement in capsid resolution allowed the side chains of the resolved density maps to be easily traced and provided good separation of the β-strands (Fig. 4B to D, and F). We then built and refined the model of an asymmetric unit and generated the whole atomic model of the HPV58 capsid (Fig. 5A and B). Structural superposition of the six models representing six nonequivalent L1 capsid proteins in one asymmetric unit showed that the core regions of the six L1s have common structures, while the N terminus and the C-terminal arms have a nonuniform projection, which is necessary for capsid assembly (Fig. 5C and D). Compared to HPV16, the structural deviation is mostly related to the surface loops (BC, DE, EF, FG, and HI), the N terminus, and the C-terminal arm regions, with root mean square deviation (RMSD) values of 1.7 Å, 1.5 Å, 2.6 Å, 1.3 Å, 1.1 Å, 4.6 Å, and 2.8 Å, respectively (Fig. 5E and F). It is worth mentioning that the relatively weak density in the N terminus and the C-terminal arm regions might cause these large deviations. Overall, our findings suggest that these seven regions contribute to HPV58 type specificity.

### Cryo-EM structures of immune complexes show distinct binding patterns.

**FIG 6:**
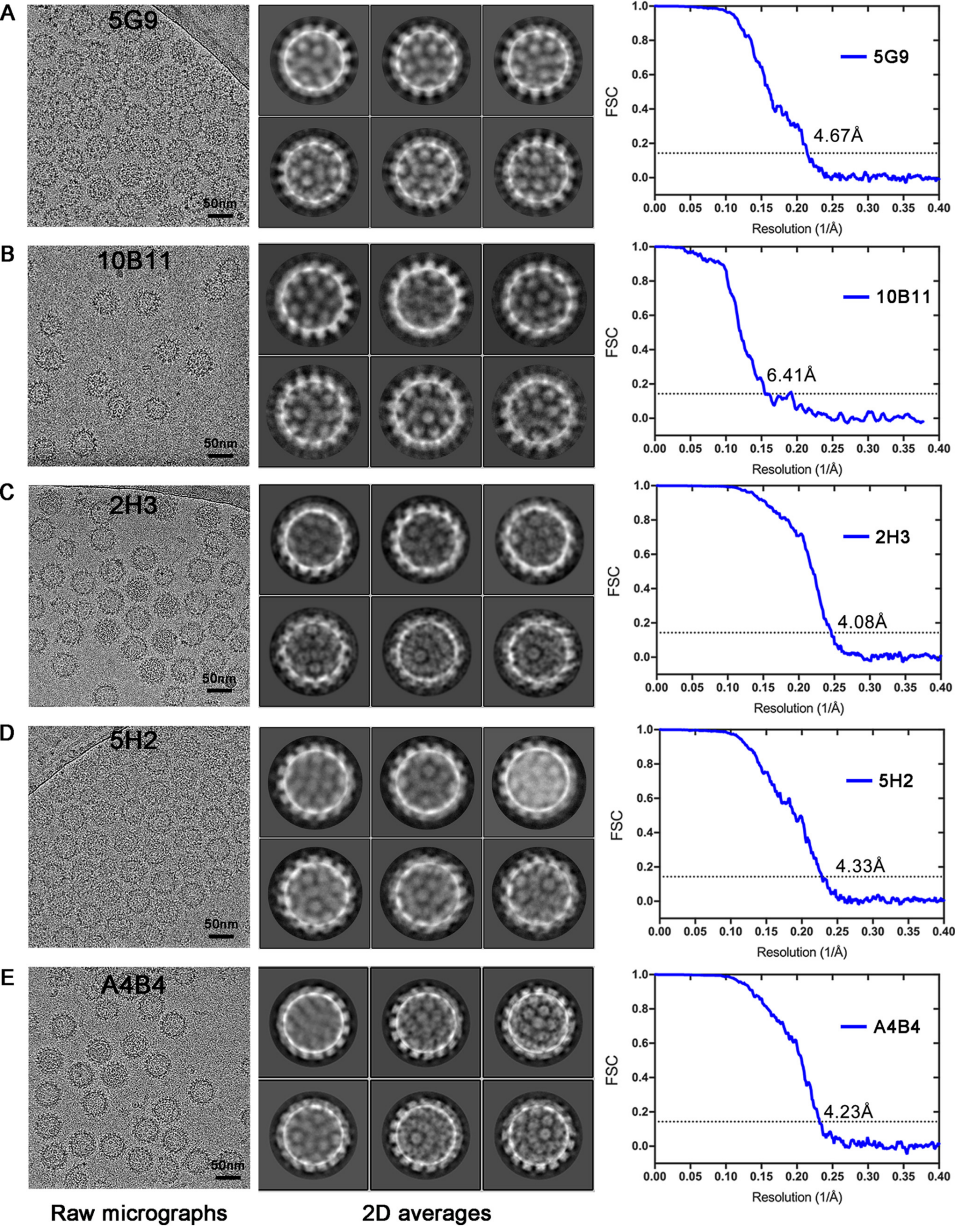
Cryo-EM reconstructions of the PsV58 immune complexes. (Left column) Representative cryo-EM micrographs of HPV58:5G9 (A), HPV58:10B11 (B), HPV58:2H3 (C), HPV58:5H2 (D), HPV58:A4B4 (E). Bar, 50 nm. (Middle column) Representative 2D class averages of HPV58:5G9 (A), HPV58:10B11 (B), HPV58:2H3 (C), HPV58:5H2 (D), and HPV58:A4B4 (E) as calculated with RELION. (Right column) Gold standard FSC curves of the icosahedral reconstructed maps of HPV58:5G9 (A), HPV58:10B11 (B), HPV58:2H3 (C), HPV58:5H2 (D), and HPV58:A4B4 (E) at resolutions of 4.67 Å, 6.41 Å, 4.08 Å, 4.33 Å, and 4.23 Å, respectively.

**FIG 7:**
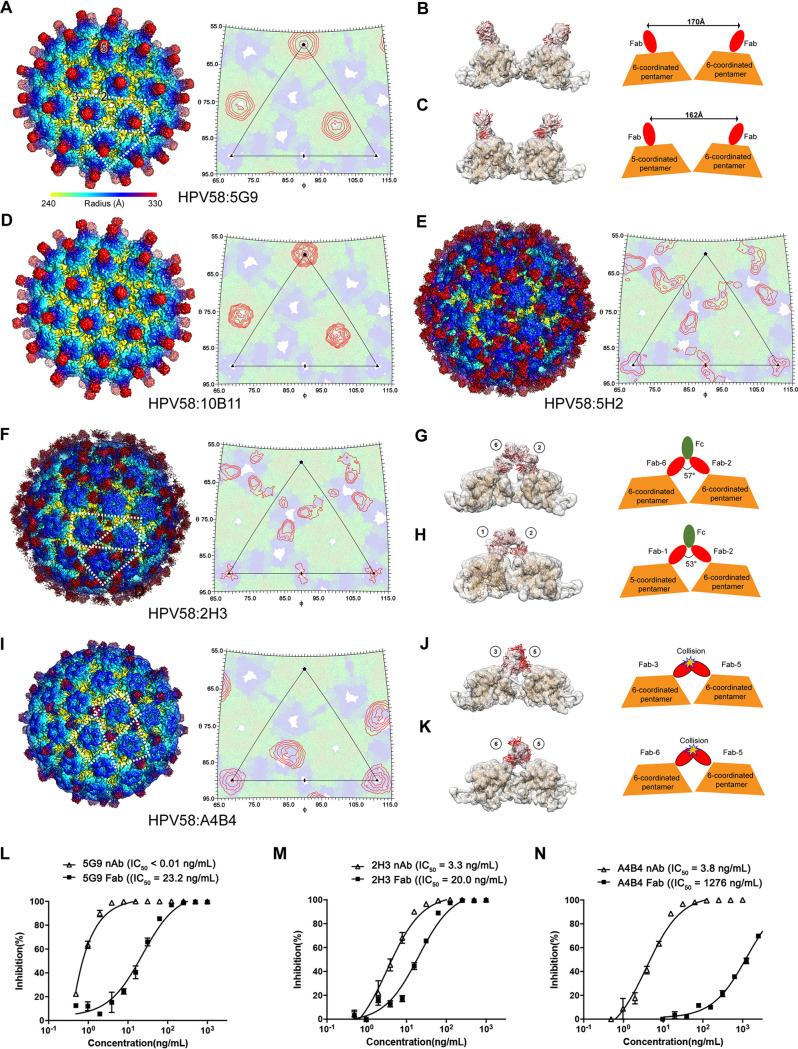
Structural analysis of the PsV58 immune complexes. (A, D, E, F, and I, left panels) Isocontoured cryo-EM maps of the PsV58 immune complexes HPV58:5G9 (A), HPV58:10B11 (D), HPV58:5H2 (E), HPV58:2H3 (F), and HPV58:A4B4 (I). Complexes are viewed along the icosahedral 2-fold axis and radially colored from 240 Å to 330 Å. Two neighboring pentamers representing unique adjacent Fab binding mode with the capsomer are boxed with white dotted rectangles. In panel A, the icosahedral asymmetric axes are indicated with the numbers 5, 3, and 2. (A, D, E, F, and I, right panels) HPV58 capsids are shown as projections of the icosahedral asymmetric units, with polar angles ϕ and θ representing the latitude and longitude, respectively. Red contour lines represent the projections of different Fabs. The extracted cryo-EM density maps of pentamers and the neighboring Fabs were fitted with HPV58 structures determined in this study and the crystal structure of Fab (PDB code 3RKD), respectively, for exploring the bivalent binding potential of 5G9 (B and C), 2H3 (G and H), and A4B4 (J and K). The schematic representations of Fabs binding on pentamers for (B, C, G, H, J and K) are on the right. (L to N) Comparative neutralization assay of nAbs in full-length and Fab forms for 5G9 (L), 2H3 (M), and A4B4 (N).

To investigate the epitope distribution of the five representative anti-HPV58 nAbs (5G9, 10B11, 2H3, 5H2, and A4B4), we prepared the Fab fragments (31) and separately incubated them with PsV58 for cryo-EM inspection and data collection. The raw cryo-EM micrographs and 2D class averages of the immune complexes revealed visible protrusions contributed by the bound Fabs (Fig. 6, left and middle). Finally, we reconstructed the immune complexes HPV58:5G9, HPV58:10B11, HPV58:2H3, HPV58:5H2, and HPV58:A4B4 at resolutions of 4.67 Å, 6.41 Å, 4.08 Å, 4.33 Å, and 4.19 Å, respectively; these were estimated where the FSC dropped below 0.143 with icosahedral symmetry (Fig. 6, right, and Fig. 7A, D to F, and I). Density maps of the five immune complexes indeed showed different binding patterns for the Fabs with the HPV58 capsid using Rivem (32) (Fig. 7A, D to F, and I, right); these differences are consistent with the above clustering analysis. We show that 5G9 and 10B11 adopt a top-center modality of binding to the HPV58 capsid, whereas 2H3 and 5H2 have a top-fringe-binding and A4B4 a fringe binding modality.

Bivalent binding of an antibody to an antigen could usually lead to stronger avidity (33). To probe possible bivalent neutralization of the representative nAbs 5G9, 2H3, and A4B4, we interrogated the geometric relationship between two neighboring Fabs and generated schematic representations for this bivalent binding potential in terms of a full-length antibody structure. For nAb 5G9, the distance between the binding sites of two adjacent Fabs is too great for a full-length antibody to bivalently bind the capsid, which was reported in other studies (12, 34, 35) (Fig. 7B and C). For nAb 2H3, we inspected the two binding Fabs astride two neighboring pentamers and found that these two binding sites allow full-length 2H3 to bivalently bind the HPV58 capsid (Fig. 7G and H). For nAb A4B4, the simultaneous binding of two adjacent Fabs would create steric hindrance and exclude any bivalent binding potential (Fig. 7J and K). We then compared the neutralization activities between full-length antibody and Fab forms and found that the Fab forms of 5G9 (Fig. 7L), 2H3 (Fig. 7M), and A4B4 (Fig. 7N) all maintained neutralization activities despite lower neutralization than full-length nAbs, especially for A4B4 with competitive binding of two neighboring Fabs (with a much higher 50% inhibitory concentration [IC_50_] of 1,278 ng/ml) (Fig. 7N).

**FIG 8:**
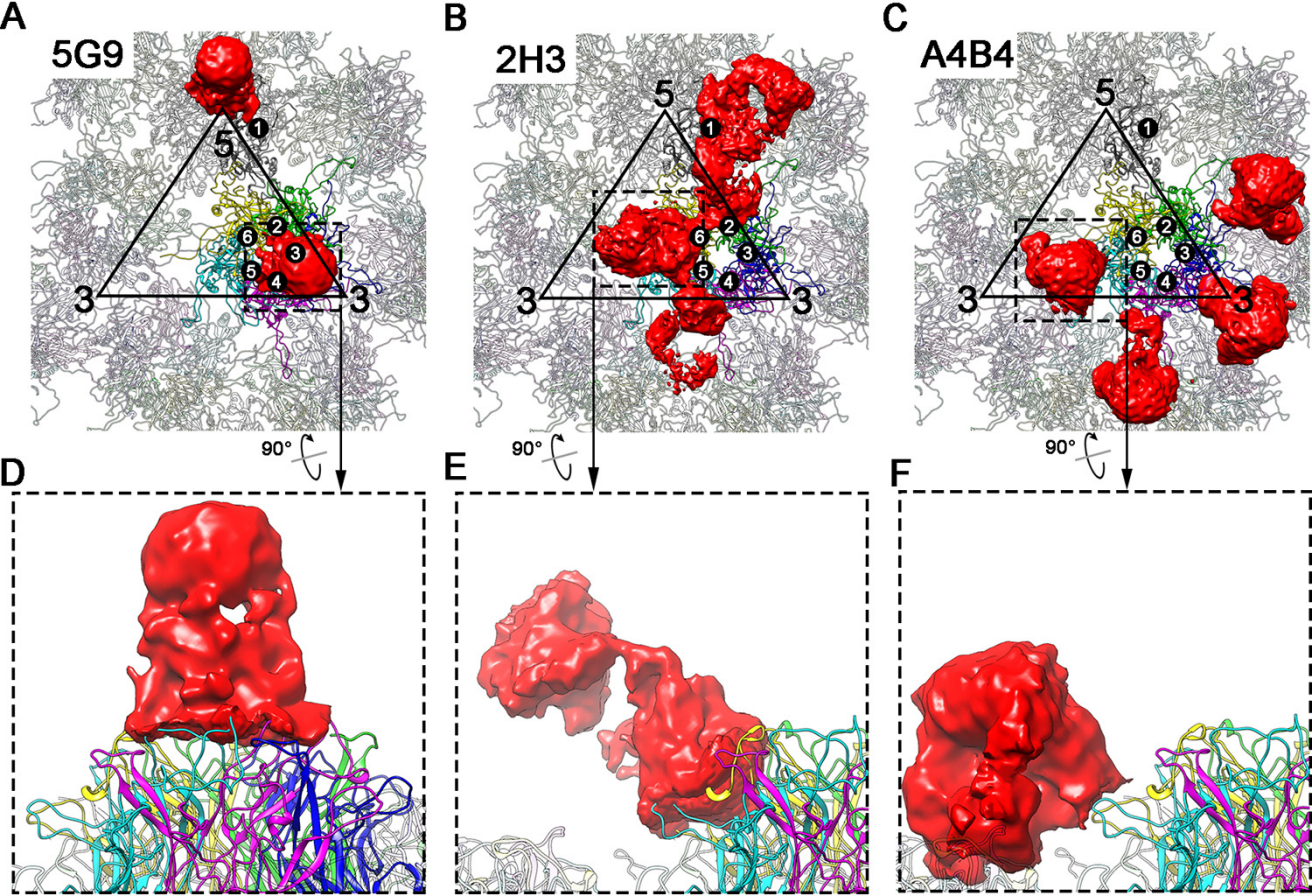
Fab occupancy analysis of three representative cryo-EM structures. (A to C) Fab occupancy on L1 monomers in icosahedral asymmetric units are shown for 5G9 (A), 2H3 (B), and A4B4 (C). Each icosahedral asymmetric unit is enclosed by a black triangle. For HPV58:5G9, each pentamer binds one 5G9 Fab, which reveals a rod-like shape; this may be due to incorrect averaging during cryo-EM reconstruction imposed by icosahedral symmetry (A). (B and C) In HPV58:2H3 and HPV58:A4B4 structures, there are four 2H3 Fabs (two with 100% occupancy and two with partial occupancy) and four smeared A4B4 Fabs within an asymmetric unit. (D to F) Close-up views show the binding profiles of Fabs 5G9 (D), 2H3 (E), and A4B4 (F). HPV58 capsids are shown in ribbon diagrams.

Considering the similar binding patterns of the two nAbs from groups C1 and C2 and the resolution of the structures of immune complexes, we selected only the following three antibodies for further interaction analysis: 5G9 (C1 group), 2H3 (C2 group), and A4B4 (C3 group). The Fab densities of these structures are smeared, which hindered their use for model building. Further analysis showed that Fab 5G9 binds vertically to the central region of each capsomer of the HPV58 capsid, and therefore, each capsomer binds only one Fab; there were no obvious variations in Fab 5G9 density when bound to pentavalent or hexavalent capsomers (Fig. 8A and D). A similar binding mode was also previously reported for HPV11 and in our previous structural study of HPV58 and HPV6 (12, 17, 36).

The density map for HPV58:2H3 showed that Fab 2H3 binds to the distal tip of each capsomer, but with a different binding occupancy within one asymmetric unit (six monomers are designated monomers 1 to 6) in pentavalent versus hexavalent capsomers. Monomers 1 and 6 have prominent densities in Fab structure, with the lowest binding hindrance at these sites. In contrast, the Fab densities for monomers 2 and 5 are smeared, possibly due to collisions with the adjacent Fabs. However, for monomers 3 and 4, there is no 2H3 Fab density near the 3-fold region (Fig. 8B and E).

Unlike the binding patterns of 5G9 and 2H3, A4B4 exclusively recognizes hexavalent capsomers and randomly binds to the fringe regions of monomers 2, 3, 4, and 5. It is presumed that the wider space between two hexavalent capsomers allows binding, in contrast to the narrower region between pentavalent and hexavalent capsomers (Fig. 8C and F).

### Subparticle reconstruction and molecular determinants of HPV immune complexes.

**FIG 9:**
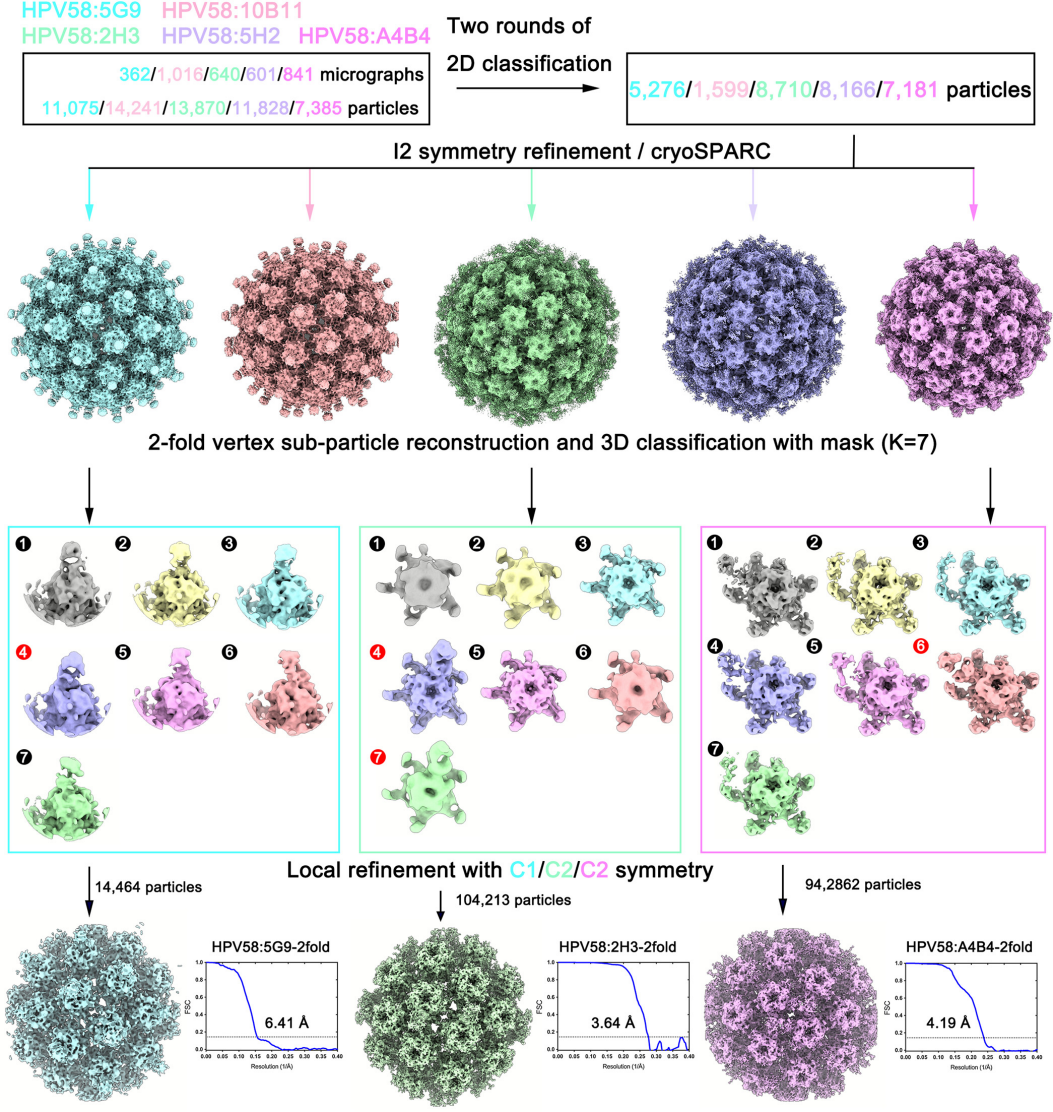
Data processing procedure for icosahedral refinement and subparticle reconstruction of 2-fold vertex regions in the PsV58 immune complex. After regular icosahedral refinement, we extracted the 2-fold vertex regions for further subparticle reconstruction, and the 3D classification was performed with masks for HPV58:5G9, HPV58:2H3, and HPV58:A4B4. Seven classes were generated, of which class 4 of HPV58:5G9, classes 4 and 7 of HPV58:2H3, and class 6 of HPV58:A4B4 were selected for local refinement for final resolution determination using cisTEM.

**FIG 10:**
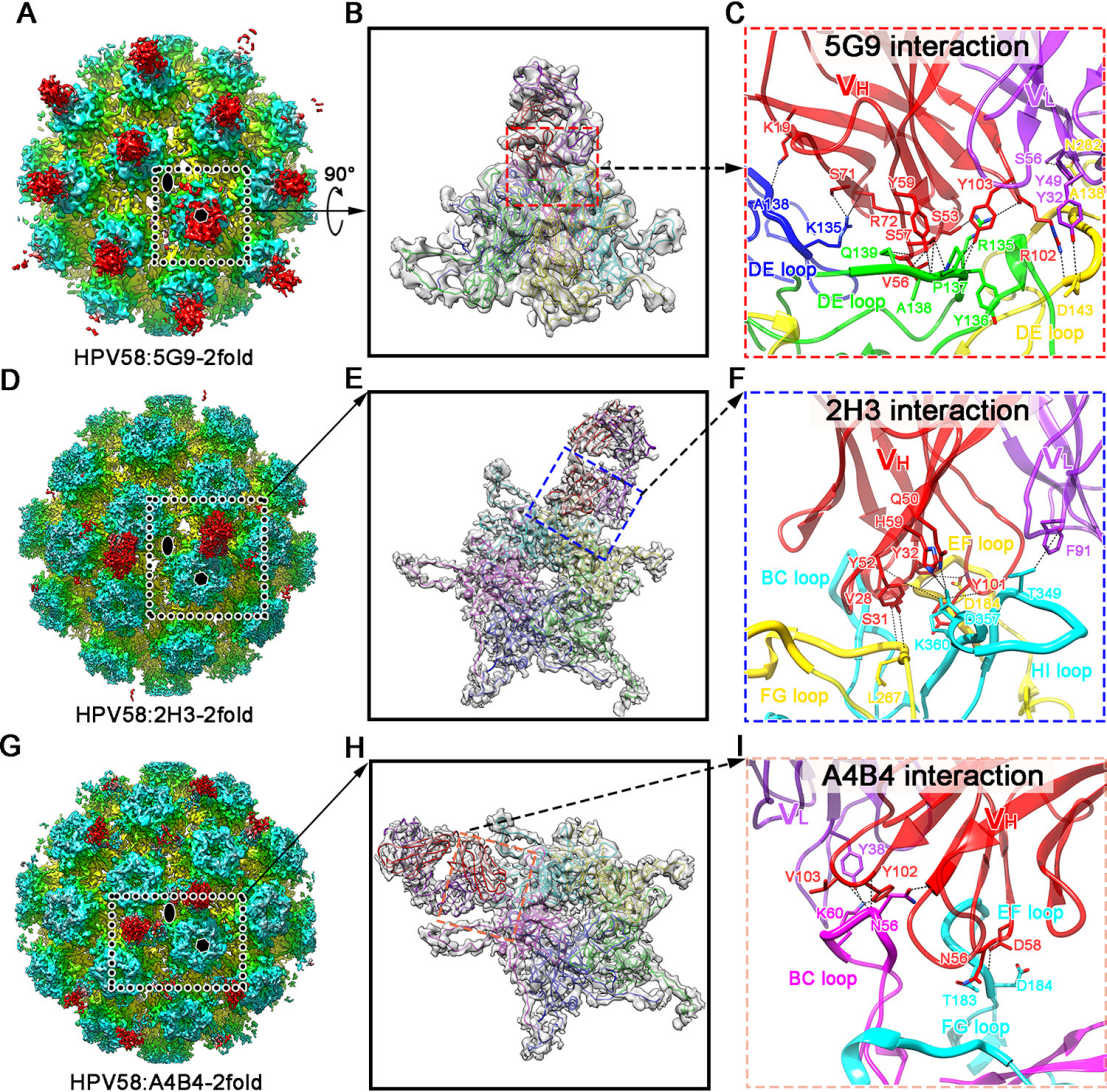
Twofold axis subparticle reconstructions and Fab-capsid interface analyses of cryo-EM structures of three representative immune complexes. (A, D, and G) Cryo-EM maps of 2-fold axis regions of HPV58:5G9 (A), HPV58:2H3 (D), and HPV58:A4B4 (G), determined at resolutions of 6.41 Å, 3.64 Å, and 4.19 Å, respectively. The oval and hexagon symbols indicate the icosahedral axes and hexamers. (B, E, and H) Close-up views of the density maps of the asymmetric units from the dashed squares in panels A, D, and G. The corresponding models in ribbon diagram are fitted. (C, F, and I) Interaction interface analysis between the capsid hexamer with Fab 5G9 (C), 2H3 (F), and A4B4 (I). The heavy chains and light chains of Fabs are colored red and purple, respectively. Potential hydrogen bonds and salt bridges are marked as black dashed lines.

To gain further insight into the molecular determinants of these different Fab binding patterns with the HPV58 capsid, we re-extracted 30 copies of the 2-fold vertex regions of the immune-complex particles as subparticles for 3D classification. The classes with obvious Fab density were selected for further refinement using cisTEM (Fig. 9). Finally, we determined the structures of HPV58:5G9-2fold, HPV58:2H3-2fold, and HPV58:A4B4-2fold at resolutions of 6.41 Å, 3.64 Å, and 4.19 Å, respectively (Fig. 9 and 10A, D, and G; Table 2). The sequences of variable regions of these Fabs are listed in Table 3. Homology models of the Fabs were generated and docked into the density maps of the 2-fold regions for further model refinement. The final models showed differences in the binding of all three nAbs to the discrete L1 monomer of the HPV58 capsid.

**TABLE 3:** Amino acid sequences of the variable domains of nAbs

nAb	Variable domain of:	
	Heavy chain	Light chain
5G9	EVMLVESGGGLVKPGGSLKLSCVASGFTFSDYAMSWGRQTPEKRLEWVATISSGGVSTYHPDSVKGRFTMSRDNAKNTLYLQMSSLRSEDTAKYYCARGGGRYAMDYWGQGTSVTVS	DVQITQSPSYLAASPGETITINCRASKSINKYLAWYQEKPGTTNKLLIYSGSTLQSGIPSRFSGRGSGTDFTLTISSLEPEDLAVYYCQQYNEYPWTFGGGTKLEIKRA
2H3	QVQLLQSGAELVRPGSSVKISCKASGYVFTSYWMHWVKQRPGQGLEWIGQIYPGDGGTHYNGNFRDKATLTADKSSSTAYMHLSSLTSEDSAVYFCARKIYDGYGFSYWGQGTLVTVS	DIQMTQSPASLSVSVGETVTITCRASENIYSNLIWYQQKQGKSPQLLVYAATNLADGVPSRFSGSGSGTQYSLKINSLQSEDFGSYYCQHFWGTPLTFGAGTKLEIKRA
A4B4	QVTLKESGPGILQPSQTLSLTCSFSGFSLSTSGMGVGWIRQPSGKGLEWLAHIWWNDDKNYHPALKSRLTISKDTSSNRVFLNIASVDTADTATYYCARIHYVTDALDYWGQGTSVTVS	DIVMSQSPSSLAVSVGEKVTMSCKSSQSLLYSSIQKNYLAWYQQKPGQSPKLLIYWASIRESGVPDRFTGSGSGTDFTLTISSVKAEDLAVYYCQRYYGYPWTFGGGTKLEIKRA

For HPV58:5G9, Fab 5G9 cross-interacted with five L1 monomers in either pentavalent or hexavalent capsomer formation, with a buried surface area of ∼1,280 Å^2^ through its heavy-chain complementarity-determining region 2 (HCDR2), HCDR3, light chain DR1 (LCDR1), and LCDR2; these four CDRs predominantly interacted with the DE loop (Fig. 10B and C; Table 4). Notably, amino acids R135, Y136, and A138 form the major interactions with 5G9, suggesting that these residues may be key residues for 5G9-based neutralization.

**TABLE 4:** Hydrogen bonding contacts between the HPV58 epitope and the Fabs

Fab	Epitope on HPV58^*a*^	Fab residue	Distance (Å)
5G9	R135^#5^ (NH1)^*b*^	R72^H^ (O)	2.80
	R135^#5^ (NH2)	S71^H^ (OG)	3.37
	A138^#5^ (O)	K19^H^ (NZ)	2.69
	R135^#6^ (NH1)	S53^H^ (OG)	3.85
	R135^#6^ (NH2)	V56^H^ (O)	2.47
	Y136^#6^ (N)	Y103^H^ (OH)	2.78
	Y136^#6^ (O)	Y103^H^ (OH)	2.30
	Y136^#6^ (O)	S57^H^ (OG)	3.14
	P137^#6^ (O)	S57^H^ (OG)	2.86
	P137^#6^ (O)	Y59^H^ (OH)	3.10
	A138^#6^ (O)	Y59^H^ (OH)	2.49
	Q139^#6^ (NE2)	G101^H^ (O)	3.18
	A138^#2^ (O)	Y49^L^ (OH)	2.72
	D143^#2^ (O)	R102^H^ (NH2)	2.90
	D143^#2^ (OD1)	Y32^L^ (OH)	3.70
	N282^#2^ (OD1)	G57^L^ (N)	3.09
	N282^#2^ (ND2)	S56^L^ (OG)	2.39

2H3	A182^#2^ (O)	V28^H^ (N)	3.77
	A182^#2^ (O)	Y32^H^ (OH)	3.0
	D184^#2^ (OD2)	Y32^H^ (OH)	3.29
	D184^#2^ (OD2)	Y101^H^ (N)	3.20
	L267^#2^ (O)	ARG102^H^ (NH1)	3.27
	G268^#2^ (O)	S31^H^ (OG)	3.04
	T349^#3^ (OG1)	F91^L^ (O)	3.12
	D357^#3^ (OD1)	Q50^H^ (NE2)	3.58
	D357^#3^ (OD1)	H59^H^ (NE2)	3.42^*c*^
	K360^#3^ (NZ)	S31^H^ (O)	2.68
	K360^#3^ (NZ)	Y101^H^ (O)	3.49

A4B4	T183^#3^ (OG1)	N56^H^ (O)	3.56
	D184^#3^ (N)	D58^H^ (OH)	3.70
	N56^#4^ (O)	V103^H^ (O)	2.92
	N56^#4^ (O)	Y102^H^ (OG)	3.68
	N56^#4^ (OD1)	G35^H^ (NZ)	2.82
	K60^#4^ (NZ)	Y102^H^ (OG)	3.19
	K60^#4^ (NZ)	Y38^L^ (OH)	2.56

a #, the residue stems from different L1 monomers, numbered according to Fig. 5B in the asymmetric unit of the HPV58 capsid.

b The atom(s) involved in hydrogen bonding contact is (are) specified in parentheses.

c This contact specifically refers to a salt bridge.

For the HPV58:2H3 immune complex, Fab 2H3 interacted with only two L1 monomers (monomers 2 and 3). However, compared with 5G9, 2H3 interacted with more loops of the PsV58 L1s—BC, EF, FG, and HI loops—with a total buried surface area of ∼1,122 Å^2^. Eleven hydrogen bonds and a salt bridge network are established in the interaction (Fig. 10E and F; Table 4). Residues A182, D357, and K360 of HPV58 are targeted by 2H3; the same residues in HPV16 and HPV18 are known to be involved in binding of the virus to the heparan sulfate cell surface receptor (37).

For HPV58:A4B4, the Fab-capsid interaction comprises 7 hydrogen bonds between the BC loop and the EF loop from two different L1 monomers (monomers 3 and 4) of the capsid and the HCDR2, HCDR3, and LCDR1 of the nAb. Compared with 5G9 and 2H3, A4B4 has the smallest buried surface area, ∼832 Å^2^ (Fig. 10H and I; Table 4). Further, the Fab A4B4 largely occupies the heparan sulfate binding region.

Taken together, our results show that the molecular determinants of three Fabs comprise the major epitopes of HPV involving the BC, DE, EF, FG, and HI loops (12–14, 17, 38, 39). Such comprehensive detail of the neutralization sites offers a new opportunity for rationally designing broad-spectrum HPV vaccines and antiviral drugs.

## DISCUSSION

In addition to HPV16 and -18, HPV58 is an important causative agent of cervical cancer according to the molecular epidemiological data, especially in Asia (4). However, the structures of the whole capsid and neutralization epitopes at the near-atomic level remain unknown. In this study, we report, for the first time, the atomic structure of the complete HPV58 and the ∼3.6- to 6.4-Å-resolution structures of the subregions of the Fab-capsid interactions, a position where density is usually of low resolution or is incorrectly averaged (Fig. 4 and 10). Given that HPV samples are generally heterogeneous, it can be difficult to ascertain an atomic-resolution structure of the whole HPV capsid. Structural ascertainment can also be hindered by the lack of a complete genome for HPV VLP/PsV/QV (11, 29). Here, we used localized reconstruction to overcome these issues and resolved the subregions surrounding the icosahedral axes to ∼3.5 Å. This, in turn, allowed us to easily trace the backbone density, even for the N terminus and C-terminal arm regions (Fig. 5 and 3C). For the Fab-PsV immune complexes, weak or incomplete Fab density in the reconstruction largely hinders the determination of epitope or key binding residues. This problem mainly stems from either occupancy hindrance of Fab binding (e.g., HPV58:2H3) or the mismatch binding of one Fab against the 5/3/2 symmetric region (e.g., HPV58:5G9 and HPV58:A4B4). Combining localized reconstruction and focused classification, we successfully pushed the local resolutions of the immune complexes, strengthened the Fab density measurements and were finally able to determine the neutralizing epitopes on the HPV58 capsid (Fig. 9). Such reconstruction will aid in the determination of interaction regions for other Fab-virion complexes.

Comprehensive neutralization epitopes are vital for an in-depth understanding of HPV infection mechanisms and for virology research. Generally, the five surface loops (BC, DE, EF, FG, and HI) of the HPV L1 protein compose the major antigenic epitopes of HPV, and this knowledge has been widely confirmed in the literature, for example, for HPV16 (38, 40, 41), HPV31 (42), HPV33 (43), and HPV6 (16, 17). In addition, the C-terminal arm of L1 serves as a neutralizing epitope, and this has been validated with loop-swapping analysis and high-resolution cryo-EM structure determination (11). Although studies have been performed for HPV58 to explore its neutralizing epitopes (18), comprehensive epitope information for HPV58 is still lacking. In this study, we generated a panel of 15 anti-HPV58 neutralizing nAbs. By combining pairwise competitive ELISA with cryo-EM, we acquired at least three representative nAb clusters and showed that these antibodies recognize different epitopes of HPV58.

We then selected three representative nAbs (5G9, 2H3, and A4B4) and explored the modes of neutralization. nAb A4B4 recognizes a linear epitope, as indicated by Western blotting, which may be derived from one of two distinct loops that constitute a conformational epitope revealed by the cryo-EM analysis. Other studies observed this kind of epitope, which was reactive in two different circumstances, i.e., the native state and the denatured state (44). nAb 2H3 recognizes an epitope involving BC, EF, FG, and HI loops of the HPV58 capsid and demonstrates the highest binding and neutralizing efficiency in the nAb panel (Fig. 10F; Table 4). The top-fringe binding pattern of 2H3 is similar to that previously reported for nAb HPV16.V5 and HPV59.28F10 (12, 39). However, the binding sites and the angle of 2H3 with the HPV58 capsid cause greater steric hindrance than nAb H16.V5. Indeed, 2H3 appears to neutralize infection by prohibiting the binding of HPV58 virions to the ECM and the cell surface; in contrast, H16.V5 blocks viral binding only to the ECM while still allowing cell surface association (19). Thus, we propose that 2H3 may serve as a physical hindrance to block the loop movements necessary during viral infection by interacting with multiple loops from two neighboring L1 monomers. Considering the immunodominance of the epitopes recognized by 2H3, this nAb may be an important candidate for assessing the integrity and antigenicity of HPV58 VLPs or future vaccine products.

Overall, this study provides detailed molecular information on the HPV58 complete virus as well as its neutralization sites. There are more than 200 distinct HPV genotypes, and there is little cross-protection offered by nAbs. Our comprehensive molecular determination of HPV58 offers new insight for the potential design of a cross-type HPV vaccine immunogen and antiviral drug and provides further knowledge for HPV-host interaction research.

## MATERIALS AND METHODS

### Generation and purification of HPV58 VLPs.

The gene encoding HPV58 L1 was synthesized (Invitrogen, Shanghai, China) according to the deposited GenBank sequence (AFS33402.1) and subsequently cloned into the pTO-T7 expression vector. The recombinant HPV58 L1 proteins were expressed in Escherichia coli after induction with 0.4 mM isopropyl-β-d-thiogalactopyranoside for 10 h at 24°C. Cells were harvested and resuspended in buffer (50 mM Tris-HCl [pH 7.2], 10 mM EDTA, 300 mM NaCl). The cells were then disrupted by sonication, and the supernatant was subjected to two rounds of chromatography, first with SP Sepharose (GE Healthcare, USA) and then with CHT II resin (Bio-Rad, USA). The HPV58 L1 proteins were then dialyzed into the buffer without the reducing agent to allow VLP self-assembly. Protein concentration was determined by bicinchoninic acid (BCA) assay, according to standard laboratory procedures. Finally, the purity and homogeneity of the HPV58 L1 proteins were inspected with SDS-PAGE and high-performance size exclusion chromatography (HPSEC).

### SDS-PAGE.

SDS-PAGE was performed according to the method of Laemmli (45), with minor modifications. Briefly, the resolved samples were mixed with reduced loading buffer, boiled for 10 min at 80°C, loaded into the wells of 10% acrylamide gels, electrophoresed, and stained with Coomassie brilliant blue.

### HPSEC.

Purified HPV58 VLPs were loaded onto a TSKgel PW5000XL 7.8-mm by 300-mm column (TOSOH, Japan), run at a flow rate of 0.5 ml/min, and separated using a 1120 compact LC high-performance liquid chromatography (HPLC) system (Agilent Technologies, Santa Clara, CA).

### Generation of murine monoclonal antibodies and preparation of the Fab fragments.

Mouse hybridomas were obtained following traditional methods, as previously described (46). Briefly, BALB/c mice were immunized via three intraperitoneal injections (0, 2, and 4 weeks) of 20 μg highly purified HPV58 VLPs adsorbed on aluminum adjuvant. A final boost of 20 μg VLPs without adjuvant was administered 72 h prior to fusion. Fused hybridomas were isolated through hypoxanthine-aminopterin-thymidine medium (Sigma, Atlanta, GA) selection, and supernatants were screened by a direct enzyme-linked immunosorbent assay (ELISA) for reactivity. Positive wells were cloned by limiting dilution. nAbs were prepared by inoculating hybridoma cells into the peritoneal cavities of pristane-primed BALB/c mice, according to a protocol approved by Xiamen University Laboratory Animal Center (XMULAC). The mouse ascites fluid was collected after 9 to 12 days. nAb samples were purified from the ascites fluid through a protein A-agarose column (Bio-Rad, Hercules, CA, USA). In brief, the ascites fluid was dialyzed against 0.2 M Na_2_HPO_4_, and the elution buffer was 0.1 M citric acid. The column flow rate was maintained at 2 ml/min, and nAbs in the eluents were detected at 280 nm. Finally, the nAbs were dialyzed into PBS and stored at −20°C. The concentration of the purified antibodies was determined using the optical density at 280 nm (OD_280_).

To obtain the Fab fragments, nAbs were digested with 1‰ (wt/wt) papain in 20 mM phosphate buffer (pH 7.0) containing 30 mM l-Cys and 50 mM EDTA for 12 h at 37°C. The reaction was quenched by the addition of 30 mM iodoacetamide. The cleaved Fab fragments were separated from the Fc fragment through a TSKgel DEAE-5PW column.

### Cell lines.

HaCaT and 293FT cells were cultured in Dulbecco’s modified Eagle’s medium (DMEM) supplemented with 10% fetal bovine serum (FBS), nonessential amino acids, and antibiotics in a humidified atmosphere at 37°C.

### Pseudovirus 58 production and purification.

The plasmids used to generate HPV58 PsVs (p58sheLL) were kindly donated by John Schiller (47) (Addgene plasmid no. 37324; RRID Addgene 37324). PsV58 was produced as described previously (48). Briefly, the HPV58 L1/L2 expression vector and pN31-EGFP were transfected into 293FT cells for 3 days. Cells were then subjected to 20% to 35% Optiprep density gradient centrifugation. The fractions containing PsV58 were collected, dialyzed against PBS buffer, and further concentrated. The concentration and integrity of PsV58 were examined by negative-staining electron microscopy.

### Pseudovirus-based neutralization assay.

The TCID_50_ (50% tissue culture infective dose) of PsV58 was measured to determine pseudovirus titers according to the classical Reed-Muench method (49). The neutralization activities of the nAbs were investigated using preattachment assays in 293FT cells. Each nAb at a starting concentration of 1,000 ng/ml was diluted over 10 points using 2-fold dilutions. Equal (50-μl) volumes of the diluted nAbs and PsV were added to the wells of a 96-well plate and incubated at 37°C for 1 h. The wells at the perimeter of the plate were not used to avoid potential plate effects.

The nAb-PsV mixture was added to 293FT cells. Plates were incubated at 37°C for 72 h. OD readings were determined using enzyme-linked immunosorbent spot (ELISPOT) assays. The neutralization data were plotted to determine the half-maximal neutralization titer.

### ELISA.

The 96-well plates were coated with antigens (300 ng/well) overnight at 4°C and then blocked with blocking buffer for 1 h at 37°C. HPV58 type-specific antibodies were added at the indicated concentrations for 45 min at 37°C. Subsequently, the plates were washed with PBS-Tween 20 (PBST) and then incubated with 100 μl horseradish peroxidase (HRP)-conjugated secondary antibodies (goat anti-mouse IgG) for an additional 45 min. The assays were developed with tetramethylbenzidine, and the reaction was terminated with 2 M H_2_SO_4_. Absorbance was measured at 450 nm with a reference wavelength of 620 nm using a Multiskan Spectrum (Thermo Fisher Scientific).

### Inhibition ELISA using rabbit serum.

A total of 8 female New Zealand White rabbits were used for immunization. Rabbits were immunized via 3 subcutaneous injections (0, 4, and 10 weeks) of highly purified HPV58 VLPs adsorbed on Freund’s adjuvant. At 2 months after immunization, rabbit standard serum was obtained from whole blood.

For the inhibition ELISA, 96-well plates were coated with HPV58 VLPs. Diluted monoclonal antibodies were added to the wells and incubated for 1 h at 37°C. The plates were washed once with PBST and then incubated with 100 μl of diluted rabbit standard serum for 30 min at 37°C. After a washing with PBST, 100 μl HRP-coupled secondary antibodies (goat anti-rabbit IgG) was added and incubated for an additional 45 min. The assays were developed with tetramethylbenzidine substrate and were stopped with 50 μl 2 M H_2_SO_4_. Absorbance was measured, and the inhibition rate was calculated.

### cELISA and cluster analysis by SPSS.

The 96-well plates were coated with 300 ng/well of HPV58 VLPs and then incubated with a saturating amount of unlabeled nAbs for 30 min at 37°C. Next, 100 μl of the diluted HRP-conjugated nAbs was added and incubated for an additional 30 min at 37°C. After color development, as described above, the OD values were measured at 450 nm with a reference wavelength of 620 nm. The value was averaged using two repeats. The control well contained only the nAb-HRP. The OD values were converted to percentage inhibition using the formula {[OD_450_ (control well) − OD_450_ (inhibited well)]/OD_450_ (control well)} × 100. Antibody cluster analyses were undertaken using SPSS (IBM SPSS statistics version 18.0; Statistical Product and Service Solutions). The hierarchical cluster analysis results are shown as dendrograms.

### 3D cryo-EM reconstruction of PsV58 and its immune complex.

Immune complexes were prepared by mixing purified PsV58 with Fab fragments in a 1.5-fold excess of the latter (equivalent to a pseudovirus-Fab molar ratio of 1:540) and then incubated at 37°C for 2 h. Aliquots (3 μl) of PsV58 samples (∼3 mg/ml) or immune complexes (∼2 mg/ml) were loaded and vitrified on holey carbon Quantifoil Cu grids (R2/2, 200 mesh; Quantifoil Micro Tools) in an FEI Mark IV Vitrobot vitrification system. Images were collected on a FEI Falcon II/III direct detector camera at a nominal magnification of ×93,000 or ×78,000 in an FEI TF30 FEG microscope at 300 kV, with underfocused settings estimated between 1.0 and 3.0 μm and an electron dose of 30 e^−^/Å^2^. Movie frame alignment and contrast transfer function (CTF) estimation of aligned micrographs were carried out with Motioncorr2.1 (50) and Gctf (51). Particles were boxed and extracted using e2boxer.py from the EMAN2.1 package (52). The initial 3D model was generated by the random model method using AUTO3DEM 4.05.2 (53). After several rounds of reference-free 2D classification and unsupervised 3D classification using RELION 2.0 (54), particles were selected for further 3D reconstruction and refinement using cisTEM (27) or cryoSPARC (55). The resolution of the final 3D density map was estimated based on the gold standard FSC curve with a cutoff of 0.143. Detailed information on the 3D reconstruction can be found in Table 2. Visualization and segmentation of the density maps were performed with Chimera (56) and ChimeraX (57). The road maps of the PsV58 immune complexes were generated by projecting the Fab densities onto the PsV58 capsid, which was depicted as a stereographic sphere using RIVEM (32).

### Localized reconstruction and refinement of vertex subparticles of the HPV58 capsid and immune complexes.

Due to the particle defocus gradient across the depth of the sample and the flexibility of the surface loops of HPV58, the resolution was limited to 4.11 Å, which is insufficient for model building. The localized reconstruction method (30) allowed us to improve the resolution by reconstructing the subparticle surrounding the 2-fold, 3-fold, and 5-fold axes of the HPV58 capsid (the defocus of each vertex subparticle was recalculated according to its location). As described above, first, we acquired the 4.11-Å-resolution structure of the HPV58 capsid through icosahedral symmetry (I2) refinement. We then determined the icosahedral orientation and center parameters for each particle image and used these values to generate the subparticles of the vertex regions, with a box size of 420 by 420 pixels at a radial distance of 260 Å from the center of the reconstructed capsid. A variable number of total subparticles for the 2-fold (403,740), 3-fold (269,160), and 5-fold (161,496) regions were then iteratively refined based on the gold standard FSC at the 0.143 criterion using cisTEM to gain final resolutions of 3.63 Å, 3.66 Å, and 3.50 Å, respectively.

For the HPV58 immune complexes, due to steric hindrance preventing two adjacent Fabs from binding the capsid simultaneously, the binding sites are not fully occupied by Fabs, so we acquired only smeared Fab densities when applying icosahedral symmetry reconstruction. Following the aforementioned subparticle reconstruction and 3D classification analysis using RELION 2.0, we selected the best classes that featured good Fab density. The total numbers of subparticles from the 2-fold regions of HPV58:5G9, HPV58:2H3, and HPV58:A4B4 (14,464, 104,213, and 94,862) were then refined to gain final resolutions of 6.41 Å, 3.64 Å, and 4.19 Å, respectively.

### Model building and refinement.

For the PsV58 structure, we used an asymmetry unit (ASU) of the HPV16 quasivirus (PDB code 5KEP) as the initial model. First, the template model was fitted into the high-resolution subparticle reconstruction density maps using Chimera. The amino acids were then corrected and adjusted manually by Coot (58). The program phenix.real_space_refine in Phenix (59) was iteratively performed to optimize the geometry of the built model. After several cycles of refinement, the refined models were merged and refined against the icosahedral reconstruction map to optimize the intermolecular clashes. The final models were validated by MolProbity (60). For the immune complexes, the initial models of the Fab fragments were generated by homology modeling using Discovery Studio software (61). Combining with the final HPV58 capsid, we fitted the models into the corresponding immune complex subparticle reconstruction density maps. The refining strategy was identical to the HPV58 capsid model refinement. All model statistics are summarized in Table 2. The PISA server (www.ebi.ac.uk/pdbe/pisa) was used to analyze the capsid-Fab interaction.

### Immunofluorescence microscopy.

Immunofluorescence microscopy was performed as previously described (62). In the cell surface binding assay, HaCaT cells were seeded onto coverslips placed in a 24-well plate and cultured overnight to ∼50% confluence. Purified PsV58 (50 ng) was preincubated with a given antibody (20 μg/ml) or BSA treatment and then added to HaCaT cells. A mouse antiserum against HPV58 VLPs served as the primary antibody and was added to the complex and incubated at 37°C for 1 h. Cells were washed and incubated in a 1:1,000 dilution of Alexa Fluor 488-conjugated donkey anti-mouse IgG to detect the antibody-bound particles. Cells were then washed again and fixed with 5% paraformaldehyde (Sigma-Aldrich) for 15 min at room temperature. After extensive washing, cells were incubated with Alexa Fluor 594-conjugated phalloidin as a secondary antibody staining step. Finally, coverslips were inverted onto a mounting solution containing DAPI (4′,6-diamidino-2-phenylindole).

In the ECM binding assay, HaCaT cells were seeded onto coverslips and cultured for 48 h. The cells were then lysed in PBS containing 10 μg/ml DNase I (Sigma-Aldrich), 0.5% Triton X-100, and 20 mM NH_4_OH at 37°C for 5 min. The HPV58 pseudovirus (50 ng) was preincubated with a specific antibody or BSA (control) at 37°C for 1 h and then incubated with ECM for another 1 h. The ECM was fixed in 4% paraformaldehyde in PBS for 15 min, washed, and then incubated with a rabbit polyclonal antibody to laminin 5 and mouse polyclonal antiserum to HPV58 VLPs. These were detected, respectively, with Alexa Fluor 594-conjugated donkey anti-rabbit IgG and Alexa Fluor 488-conjugated donkey anti-mouse IgG. Coverslips were then mounted in DAPI-containing mounting solution. All immunofluorescence microscopy images were acquired with a Zeiss LSM 780 confocal system and processed with Adobe Photoshop software. The fluorescence of PsV attachment was calculated in 10 random inspection views. Data were analyzed by unpaired Student's *t* test. Statistical significance was determined as a *P* value of <0.05 using GraphPad Prism 7.0.

### Data availability.

The cryo-EM density maps and corresponding atomic coordinates have been deposited in the Electron Microscopy Data Bank (EMDB) and Protein Bank (PDB), respectively. The accession codes are as follows: HPV58 pseudovirus capsid, EMD-30781 and PDB code 7DN5; HPV58-2fold, EMD-30768; HPV58-3fold, EMD-30769; HPV58-5fold, EMD-30770; HPV58:5G9, EMD-30772; HPV58:5G9-2fold, EMD-30786 and PDB code 7DNK; HPV58:10B11, EMD-30773; HPV58:2H3, EMD-30774; HPV58:2H3-2fold, EMD-30783 and PDB code 7DNH; HPV58:5H2, EMD-30777; HPV58:A4B4, EMD-30780; HPV58:A4B4-2fold, EMD-30787 and PDB code 7DNL.
